# Effect of oral statin use on mitomycin-C augmented trabeculectomy outcomes

**DOI:** 10.1371/journal.pone.0245429

**Published:** 2021-01-15

**Authors:** Abhibol Inobhas, Sunee Chansangpetch, Anita Manassakorn, Visanee Tantisevi, Prin Rojanapongpun

**Affiliations:** 1 Department of Ophthalmology, Faculty of Medicine, Chulalongkorn University, Bangkok, Thailand; 2 King Chulalongkorn Memorial Hospital, Thai Red Cross Society, Bangkok, Thailand; 3 Glaucoma Research Unit, Chulalongkorn University, Bangkok, Thailand; Faculty of Medicine, Cairo University, EGYPT

## Abstract

**Purpose:**

The effect of statins on wound healing is controversial, and their effect on trabeculectomy outcomes remains unclear. This study aimed to examine the relationship between oral statin use and trabeculectomy outcomes.

**Methods:**

Medical records of patients who underwent primary mitomycin-C augmented trabeculectomy with 2 years of follow-up were reviewed. Pre- and postoperative intraocular pressures (IOP) and numbers of medications, subconjunctival 5-fluorouracil (5-FU) injections, and bleb-needling procedures were compared between statin users and nonusers. Failure was defined as an eye that failed to achieve a 20% lowering of IOP from baseline or had an IOP > 21 mm Hg, as well as an eye that required further surgical intervention, developed hypotony, or had no light perception visual acuity.

**Results:**

In total, 158 subjects were enrolled, with 47 eyes from statin users and 111 eyes from statin nonusers. The 24-month cumulative probability of failure was 78.7% for statin users and 60.4% for nonusers (*P* = .013). Cox proportional-hazards modeling showed a significantly higher hazard risk in statin users (adjusted hazard ratio 1.61, *P* = .026). There were no significant between-group differences in mean IOPs or number of medications (both *P* > .05) at 24 months. Multivariable Poisson regression analysis that statin use was associated with increased numbers of 5-FU injections (*P* = .014) and bleb-needling procedures (*P* = .031).

**Conclusions:**

This study demonstrated that oral statin use was associated with higher rates of trabeculectomy failure and increased numbers of 5-FU injections and bleb-needling procedures.

## Introduction

Glaucoma is a progressive optic neuropathy that causes irreversible blindness if not treated appropriately. Treatment approaches for this disorder are aimed at controlling intraocular pressure (IOP), a major modifiable factor related to disease progression. Current treatment modalities include topical medications, laser therapies, and surgical procedures [1, 2].

Trabeculectomy is now the most widely performed surgical procedure for treatment of medically uncontrolled glaucoma in adults. While wound healing is required for other types of surgery, trabeculectomy is unique because its success is linked to interruption of this wound-healing process in order to maintain patency of the new filtration pathway. This goal makes subconjunctival scarring one of the most challenging issues facing clinicians after trabeculectomy [3, 4].

Statins, competitive inhibitors of 3-hydroxy-3-methylglutaryl coenzyme A (HMG-CoA) reductase, are generally used to decrease plasma low-density lipoprotein cholesterol levels and to prevent cardiovascular disease-related morbidity and mortality. In addition to their lipid-lowering properties, statins have been shown to have a myriad of other effects on the body, including antioxidative, antiproliferative, angiogenic, and anti-inflammatory effects. The use of statins has increased sharply in recent years, and they are now one of the most commonly prescribed classes of medications worldwide [5, 6].

Researches have shown that statin use is associated with a lower risk of primary open-angle glaucoma (POAG), a lower progression rate of visual field defects [7, 8], and a significant increase in aqueous outflow through relaxation of the trabecular meshwork and the ciliary body muscle [9]. Reports have also suggested that statins play a role in fibrosis, which theoretically could affect the wound-healing process following trabeculectomy [10, 11]. This study aimed to evaluate the effect of oral statin use on patient outcomes after trabeculectomy.

## Methods

The protocol for this retrospective, comparative, interventional study was approved by the Institutional Review Board of the Faculty of Medicine at Chulalongkorn University. The requirement for written informed consent was waived due to the retrospective nature of the study. Medical records of patients who underwent primary mitomycin-C (MMC)-augmented trabeculectomy and completed 2 years of follow-up were reviewed.

### Study population

Patients were considered to be eligible for inclusion if they met the following criteria: 1) were at least 20 years of age, 2) met the International Society of Geographical and Epidemiological Ophthalmology’s diagnostic criteria for POAG, primary angle-closure glaucoma (PACG), or secondary glaucoma [12], and 3) had regular follow-up evaluations for 24 months after primary trabeculectomy. If both eyes were treated with primary trabeculectomies, only the right eye was included in the study.

Patients were excluded from this study if they met any of the following criteria: 1) previous history of a trabeculectomy, 2) diagnosis of childhood glaucoma, 3) concurrent performance of trabeculectomy and other procedures, including cataract surgery or glaucoma drainage device implantation, 4) significant conjunctival pathology over the superior bulbar area, 5) active ocular inflammation, or 6) previous intraocular surgery within 3 months prior to trabeculectomy.

### Study variables

The following data were collected for all study participants: baseline characteristics, pre- and postoperative IOPs, visual acuity, Humphrey visual field mean deviation, cup-to-disc ratio, and number of glaucoma medications. The total number of subconjunctival 5-fluorouracil (5-FU) injections and bleb-needling procedures performed after trabeculectomy were also recorded.

Information about patient statin use versus nonuse was also collected. Statin users were defined as patients who regularly took any type of oral statin, starting at any time before primary trabeculectomy and continuing for at least 24 months after surgery. Subsequently, study variables were compared between statin users versus nonusers.

### Study intervention and follow-up

All trabeculectomies were performed by certified glaucoma specialists using the same fornix-based approach incision and trapezoid scleral flap technique. Subconjunctival space was undermined to create the area of reservoir in a fan-shaped fashion as far posterior and as large horizontal as possible. A surgical sponge (Weck-Cel; Alcon Surgical, Fort Worth, TX, USA) soaked in 0.3 mg/mL MMC was placed into the prepared subconjunctival area and removed after 3 to 4 minutes, after which the area was irrigated with a balanced salt solution. Topical corticosteroids were prescribed for use four to six times daily during the initial postoperative period and then tapered off within the following 2 to 3 months.

All patients were scheduled for regular early postoperative follow-up at one day, one week, and then every two weeks or timely at surgeon’s consideration after the procedure until discontinuation of topical corticosteroid use. Unexpected or undesired events, including a postoperative IOP rise or hypotony, were evaluated at nonroutine follow-up visits. After the initial phase of follow-up, patients were evaluated regularly at the discretion of their surgeons.

Postoperative bleb morphology was assessed using the Indiana Bleb Appearance Grading Scale (IBAGS) [13], which is based on the presence of vascularity, bleb height, and bleb extent. Postoperative subconjunctival injections of 5 mg of 5-FU were performed if blebs demonstrated IBAGS vascularity grades of V3 to V4 (moderate to extensive bleb vascularity). Bleb-needling procedures were performed if the IOP was over the target value customized to the patient and if there was episcleral fibrosis or bleb encapsulation. Bleb needling was performed with or without subconjunctival 5-FU injections depending on bleb vascularity.

### Outcomes

This study’s primary outcome was treatment failure, which was defined as any eye with an inadequately lowered IOP after 3 months (i.e. failure to lower the IOP by 20% from baseline or an IOP > 21 mm Hg) or any eye that required further glaucoma-related surgical interventions, developed hypotony (IOP ≤ 5 mmHg), or had no light perception. Failure was analyzed and reported in two ways: (1) ‘cumulative failure,’ or the cumulative number of failure events that occurred at any timepoint within 24 months and (2) ‘failure at 24 months,’ or the number of eyes that met the failure definition at the final timepoint of the study regardless of antiglaucoma medication use.

Secondary outcomes included other clinical characteristics at 24 months, including visual acuity, IOP, number of glaucoma medications, and total numbers of 5-FU injections and needling procedures required during follow-up.

### Statistical analysis

Statistical analyses were performed using Stata 13.0 (StataCorp, College Station, TX). Means with standard deviations (SD) were calculated for continuous variables, and frequencies with percentages were tabulated for categorical variables. The Student's *t*-test or the Mann-Whitney *U* test were used to compare continuous variables. The chi-squared test or the Fisher's exact test were used to compare categorical variables.

Survival analysis was performed using the Kaplan-Meier method. The log-rank test was used to compare cumulative failure rates between groups. The association between statin use and the cumulative failure rate was assessed using a Cox proportional-hazards regression model.

A logistic mixed-model regression was used to investigate the association between statin use and the failure rate at 24 months. Multivariable Poisson regression models were then built to evaluate the effect of statin use on the total numbers of 5-FU injections and bleb-needling procedures. All multivariable models were adjusted for age, gender, lens status, history of diabetes mellitus, and glaucoma diagnosis. Logistic regression analyses were modeled with the follow-up time. A *P*-value of < .05 was considered statistically significant.

## Results

A total of 158 eyes of 158 patients were eligible for this study (Table 1), with 47 statin users and 111 nonusers. Eighty-three (53%) patients were male, and the mean (SD) age of patients was 59.6 (13.9) years. Most eligible eyes were phakic (115/158, 73%). Nearly half of patients in both groups (69/158, 44%) were diagnosed with secondary glaucoma. There was no statistically significant difference in the type of secondary glaucoma between statin users and nonusers (*P* = .33) (S1 Table). The mean (SD) preoperative IOPs were 26.3 (12.4) mm Hg in statin users and 27.4 (12.2) mm Hg in nonusers. There were no statistically significant differences in sex, laterality, lens status, type of glaucoma, visual acuity, IOP, number of medications, cup-to-disc ratio, or visual field mean deviation between the groups before the primary trabeculectomy surgery. Moreover, there was no significant difference in the median of duration of MMC in both groups [median (IQR): statin users 3 (3–3.5) minutes, statin nonusers 3 (3–3.5) minutes, *P* = .79].

**Table 1:** Baseline patient characteristics.

Characteristics	Statin nonusers (N = 111)	Statin users (N = 47)	*P*-value
Sex*			.65
Male	57 (51%)	26 (55%)	
Female	54 (49%)	21 (45%)	
Age† (year)	57.2 (14.3)	65.4 (11.2)	< .001
Laterality*			.36
Right	72 (65%)	34 (72%)	
Left	39 (35%)	13 (28%)	
Lens status‡			.02
Phakic	87 (78%)	28 (60%)	
Pseudophakic	24 (22%)	19 (40%)	
Diabetes mellitus*	15 (14%)	26 (55%)	< .001
Diagnosis*			.75
Primary open-angle glaucoma	33 (30%)	15 (32%)	
Primary closed-angle spectrum	30 (27%)	11 (23%)	
Secondary glaucoma	48 (43%)	21 (45%)	
Intraocular pressure† (mm Hg)	27.4 (12.2)	26.3 (12.4)	.61
Number of medications†	3.8 (1.0)	3.9 (0.8)	.35
Mean deviation§ (dB)	-21.45	-19.65	.43
	(-29.83 to -9.44)	(-21.86 to -10.02)	
	N = 24	N = 13	
Visual acuity§ (decimal)	0.29 (0.01 to 0.67)	0.29 (0.01 to 0.50)	.67
Cup-to-disc ratio§	0.8 (0.7 to 0.9)	0.7 (0.5 to 0.85)	.08

* Data presented as n (%); *P*-value was obtained from chi-squared test.

† Data presented as mean (standard deviation); *P*-value was obtained from Student’s *t*-test.

‡ Data presented as n (%); *P*-value was obtained from Fisher’s exact test.

§ Data presented as median (IQR); *P*-value was obtained from Mann-Whitney *U* test.

**Fig 1:**
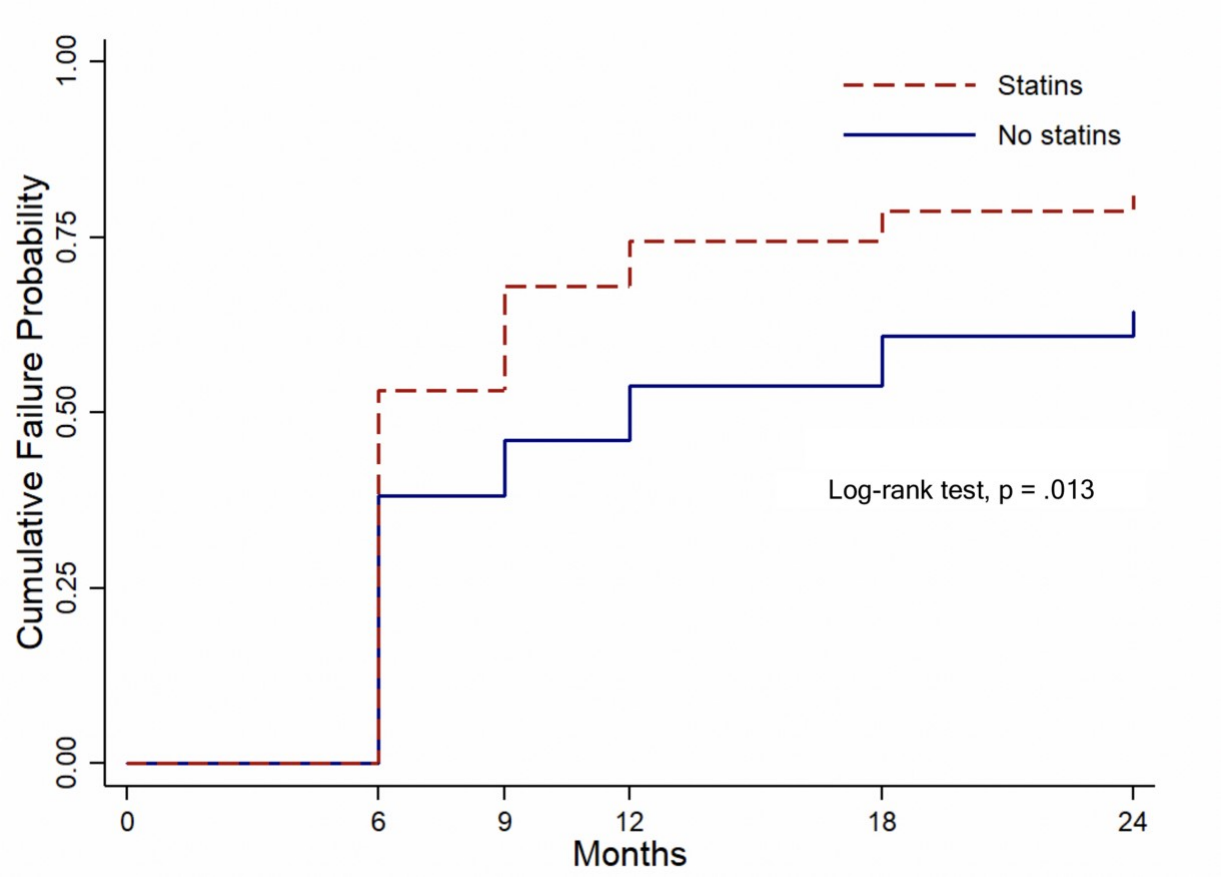
Kaplan-Meier survival analysis with log-rank test. The analysis shows that the statin user group had a significantly higher cumulative probability failure than the nonuser group (*P* = .013).

Kaplan-Meier survival analysis with the log-rank test (Fig 1) showed that the statin user group had a significantly higher cumulative failure probability than the nonuser group (*P* = .013), with a 24-month cumulative failure probability of 78.7% in statin users and 60.4% in nonusers. Identified causes for cumulative failure in both patient groups are shown in Table 2. The Cox proportional-hazards model revealed that the unadjusted hazard ratio for statin users was 1.51 (95% confidence interval (CI): 1.02–2.24, *P* = .042). The multivariable model showed that the adjusted hazard ratio for statin users was 1.61 (95% CI: 1.06–2.45, *P* = .026).

**Table 2:** Causes of 24-month cumulative failure after trabeculectomy in statin users and nonusers.

	Statin nonusers (N = 111)	Statin users (N = 47)
Inadequate lowering of intraocular pressure	43 (38.7%)	23 (48.9%)
Reoperation for glaucoma	21 (18.9%)	14 (29.8%)
Hypotony	3 (2.7%)	1 (2.1%)
No light perception	3 (2.7%)	4 (8.9%)

Failure rate at 24 months was 46.8% in statin users and 39.6% in nonusers. There were no statistically significant differences in visual acuity, IOP, or number of medications between the groups at 24 months (*P* = .62, *P* = .15, and *P* = .99, respectively) (Table 3). Logistic mixed-model regression analysis showed an increased risk of failure at 24 months for statin users using both univariate (odd ratio [OR] = 3.72, 95% CI: 1.10–12.62, *P* = .035) and multivariate models (adjusted OR = 5.89, 95% CI: 1.44–23.94, *P* = .013).

**Table 3:** Clinical outcomes in statin users and nonusers at 24-month follow-up after trabeculectomy.

	Statin nonusers (N = 111)	Statin users (N = 47)	*P*-value
Visual acuity* (decimal)	0.29 (0.01 to 0.67)	0.4 (0.005 to 0.67)	.62
Intraocular pressure† (mm Hg)	12.7 (5.2)	14.4 (8.9)	.15
Number of glaucoma medications†	0.95 (1.3)	0.96 (1.3)	.99
Mean deviation* (dB)	-16.31 (-27.5 to -9.84) (N = 14)	-12.69 (-20.71 to -0.56) (N = 11)	.34
Cup-to-disc ratio*	0.85 (0.7 to 0.95)	0.7 (0.5 to 0.85)	.28

* Data presented as median (IQR); *P*-value was obtained from Mann-Whitney *U* test.

† Data presented as mean (standard deviation); *P*-value was obtained from Student’s *t*-test.

The mean (SD) number of 5-FU injections was 1.87 (2.1) in statin users and 1.21 (1.7) in statin nonusers. Multivariable Poisson regression analysis of the number of 5-FU injections indicated that statin users had a significantly higher number of 5-FU injections at 24 months [unadjusted incidence rate ratio (IRR) = 1.55, 95% CI: 1.19–2.03, *P* = .001; adjusted IRR = 1.59, 95% CI: 1.08–2.08, *P* = .014).

The mean (SD) number of bleb-needling procedures was 0.66 (1.0) in statin users and 0.32 (0.7) in statin nonusers, with a significantly higher number of bleb-needling procedures performed in statin users than in nonusers. (unadjusted IRR = 2.09, 95% CI: 1.29–3.39, *P* = .003 and adjusted IRR = 1.91, 95% CI: 1.06–3.45, *P* = .031).

Regarding bleb morphology, the bleb extent and height at 3 months post-operation according to IBAGS grading showed no significant difference between both groups (extent *P* = .86 and height *P* = .19). Statin users showed greater vascularity than nonusers (*P* < .001). Representative images of bleb vascularity grades of V2 to V4 according to IBAGS grading are shown in S1 Fig.

In order to control for the effects of age, an additional analysis involving 2:1 age matching analysis was performed. The data from 80 statin nonusers and 40 users showed the same results. The 24-month cumulative failure probability was significantly higher in statin users (77.5%) than in nonusers (62.5%) (*P* = .048). The Cox proportional-hazards model revealed that the hazard ratio for statin users was 1.44 (95% CI: 0.92–2.24, *P* = .11). Similarly, the multivariable Poisson regression analysis also showed a significantly higher number of 5-FU injections and bleb-needling procedures in statin users (IRR = 1.76, 95% CI: 1.31–2.37, *P* <0.001; IRR = 1.93, 95% CI: 1.14–3.27, *P* = .015, respectively).

## Discussion

Our study revealed that statin use significantly increased the hazard risk of trabeculectomy failure and significantly increased the failure rate at 24 months. There were no statistically significant differences in the mean IOP, visual acuity, or number of medications between statin users and nonusers at 24 months. Statin users, however, underwent significantly more 5-FU injections and bleb-needling procedures than nonusers.

The present study identified higher trabeculectomy failure rates, both for cumulative failure and for failure at 24 months, than other clinical trials [14, 15]. Our cumulative probability of failure during 2 years of follow-up was 78.7% in statin users and 60.4% in nonusers. By contrast, in the Tube Versus Trabeculectomy Study, Gedde et al. reported a 46.9% failure rate in their trabeculectomy group during 5 years of follow-up [16]. Our higher failure rate may be explained by the retrospective nature of our study, which precluded assessment of washed-out IOPs and allowed only medicated IOP values to be obtained. Since mean preoperative IOPs are lower when using only medicated IOP data, a greater number of eyes failed to achieve a 20% IOP reduction from baseline in our study.

Inadequate IOP reduction was the most common cause of failure in both groups, though more commonly in statin users. The success of trabeculectomy at reducing IOP is linked to an effective interruption of the wound-healing response in order to maintain patency of the new filtration pathway. In a simplistic sense, wound healing after trabeculectomy may be understood by dividing the process into three distinct phases, inflammatory, proliferative, and remodeling phases [4, 17]. Previous studies have suggested that statin use may be linked to an acceleration of this wound-healing process [18] and to excessive fibrosis formation [10, 11], which may explain the higher failure rate in the statin group observed in our study.

The inflammatory phase of wound healing is characterized by an influx of inflammatory cells, cytokines, and growth factors. In the early stages, this phase is controlled by immediate leakage of blood components from incised vascular tissue. Several chemical factors, including thrombin, thromboxane A_2_, platelet-derived growth factor (PDGF), vascular endothelial growth factor (VEGF), interleukin (IL) -1, transforming growth factor (TGF-β), and interferon-γ (IFN-γ) are also secreted, triggering the onset of the next phase [19–21]. Previous studies have shown that statins induced proinflammatory responses by increasing the number of T lymphocytes secreting IFN-γ and by stimulating the secretion of IL-1ß, a member of the IL-1 family of cytokines, by monocytes. Moreover, studies have demonstrated that caspase-1, which is required for the processing and secretion of IL-1ß, was activated by statins [22, 23].

In the proliferative phase, several processes are stimulated, including re-epithelialization, angiogenesis, and fibroblast activity. PDGF and TGF-β are key profibrogenic cytokines implicated in the recruitment and differentiation of fibroblasts into myofibroblasts. Akershoek et al. [24] demonstrated an acceleration of the healing process in full-thickness burn wounds in patients receiving atorvastatin treatment. These authors hypothesized that the shortened healing time in atorvastatin users was related to an earlier transition from the inflammatory to the proliferative phase of wound healing and to a faster resolution of myofibroblast presence in wounds.

During the proliferative phase, angiogenesis occurs concurrently with release of VEGF from platelets and macrophages [4, 17]. Numerous studies have demonstrated effects of statins on angiogenesis, including upregulation of endothelial nitric oxide synthase, stimulation of the intracellular serine/threonine kinase Akt signaling pathway in endothelial cells, and promotion of the migration and differentiation of endothelial progenitor cells [25–27]. In 2012, a histopathology study demonstrated that topical simvastatin accelerated wound healing in diabetic mice via promotion of angiogenesis and lymphangiogenesis [18], which supported previous *in vitro* studies showing the marked angiogenic effects of statins on vascular endothelial cells [28, 29]. In addition, statins have been reported to augment VEGF expression in osteoblastic cells and to stimulate retinal microvasculature angiogenesis [30, 31]. The presence of VEGF in trabeculectomy blebs has also been shown to be associated with blood vessel growth and fibrosis [32], a finding that clarified the role of anti-VEGF therapies during wound-healing modulation after glaucoma-filtering surgery [33]. In our study, statin use increased the number of 5-FU injections that were required. Since 5-FU injections were performed when blebs were highly vascularized, we postulate that the higher number of 5-FU injections in the statin group may reflect augmentation of VEGF expression by this class of drugs.

In 2012, Xu and colleagues [34] demonstrated that statin use was associated with interstitial lung abnormalities, increasing a patient’s susceptibility to interstitial lung disease. Lungs from mice treated with statins and bleomycin were shown to have increased lung fibrosis and collagen deposition compared to lungs of mice exposed to bleomycin alone. The statin and bleomycin group also had significant increases in expression of IL-1β and IL-18 in bronchoalveolar lavage fluid and in lung tissue, which was correlated with increased caspase-1 activation. Furthermore, another study described pulmonary side effects from statins, including the development of hypersensitivity pneumonitis and fibrotic nonspecific interstitial pneumonia [10]. Although similar studies have yet to be conducted in the human eye, these findings suggest that there may be a correlation between statin use and fibrosis formation after trabeculectomy.

While these previous studies have demonstrated possible negative effects of statin use on bleb outcomes, other studies have contradicted our findings by demonstrating that statins may actually inhibit postoperative scarring after glaucoma-filtering surgery. In 2008, Mayer-ter-Venh et al. [35] reported that lovastatin inhibited TGF-β-induced myofibroblast transdifferentiation in human tenon fibroblasts, which is crucial for activation of fibroblasts in the proliferative phase and for proper extracellular matrix replacement during the remodeling phase [36, 37]. In addition, Park and colleagues [38] demonstrated lovastatin’s antifibrotic effect by showing reduced myofibroblast transdifferentiation after glaucoma-filtering surgery in a rabbit model.

We hypothesize that the contradictory findings in these prior studies may be related to the types of statins used and to different routes of administration. Moreover, wound healing is known to be a multifactorial and complicated process in which individuals may mount responses differently. Another possible explanation is that statins may have biphasic, dose-dependent effects on angiogenesis, promoting the response at lower doses and inhibiting it at higher doses, similar to what has been proposed for endothelial cell proliferation, migration, differentiation, and VEGF release [26, 39]. Therefore, different or unknown doses of statins in our study and others might have influenced these processes differently. The relationships between the types and dosages of statins used and the wound-healing process still need to be explored in future studies.

It is important to note that this study had limitations. First, since it was a retrospective study, we could not perform objective assessments of bleb morphology, such as bleb imaging, which would have helped to verify the presence of fibrosis. Second, the type and dosage of statins used by patients was not systematically recorded. Third, the study was conducted in a uniformly Thai population, and therefore, its applicability to other ethnicities, especially non-Asian ethnicities, needs to be validated. Fourth, the majority of our study patients had advanced glaucoma. The results may not, therefore, be generalizable to patients with other degrees of glaucoma severity. Additionally, bleb grading, 5-FU injections, and bleb-needling procedures were performed at doctors’ discretion. It should be noted, however, that all the doctors who were making these decisions and performing these procedures were glaucoma specialists practicing under the same guidelines outlined by our institution. Lastly, we were unable to evaluate the duration of topical antiglaucoma medication usage before glaucoma-filtering surgeries, which has been shown to have a deleterious effect on trabeculectomy outcomes [40]. In the future, prospective studies using bleb imaging to better understand the effect of statin use on scar formation following trabeculectomy are warranted.

In conclusion, this study demonstrated that oral statin was associated with higher rates of trabeculectomy failure. Statin use also increased the number of 5-FU injections and bleb-needling procedures required in the post-trabeculectomy period. Although further investigation is necessary, this study suggests that there may be a link between oral statin use and postoperative scarring after glaucoma-filtering surgery.

## Supporting information

S1 Table Type of secondary glaucoma in statin users and nonusers. (PDF)

S1 Fig Images showing bleb vascularity grades of V2 (mild vascularity), V3 (moderate vascularity) and V4 (extensive vascularity) according to Indiana Bleb Appearance Grading Scale (IBAGS). (TIFF)

S1 File (XLSX)
