# Supplementary material for: Effect of oral statin use on mitomycin-C augmented trabeculectomy outcomes
Source: PLoS One. 2021 Jan 15;16(1):e0245429. doi: 10.1371/journal.pone.0245429 (PMC7810309; doi:10.1371/journal.pone.0245429)
Supplement: S1 Table — (PDF) [file pone.0245429.s001.pdf]

S1 Table. Type of secondary glaucoma in statin users and nonusers.

**Type of secondary glaucoma**	**Statin nonusers (N = 111)**	**Statin users (N = 47)**
Associated with intraocular inflammation (NVG + uveitis glaucoma)	25 (22.5%)	17 (36.2%)
Associated with surgery or trauma (cataract surgery, retina surgery, trauma)	11 (9.9%)	2 (4.2%)
Associated with corneal disease (ICE syndrome, corneal transplant)	4 (3.6%)	1 (2.1%)
Pseudoexfoliation	2 (1.8%)	0 (0%)
Steroid-induced glaucoma	4 (3.6%)	0 (0%)
Associated with increased episcleral venous pressure (TRO)	2 (1.8%)	1 (2.1%)
