# Supplementary figures and images for: Effect of oral statin use on mitomycin-C augmented trabeculectomy outcomes
Source: PLoS One. 2021 Jan 15;16(1):e0245429. doi: 10.1371/journal.pone.0245429 (PMC7810309; doi:10.1371/journal.pone.0245429)

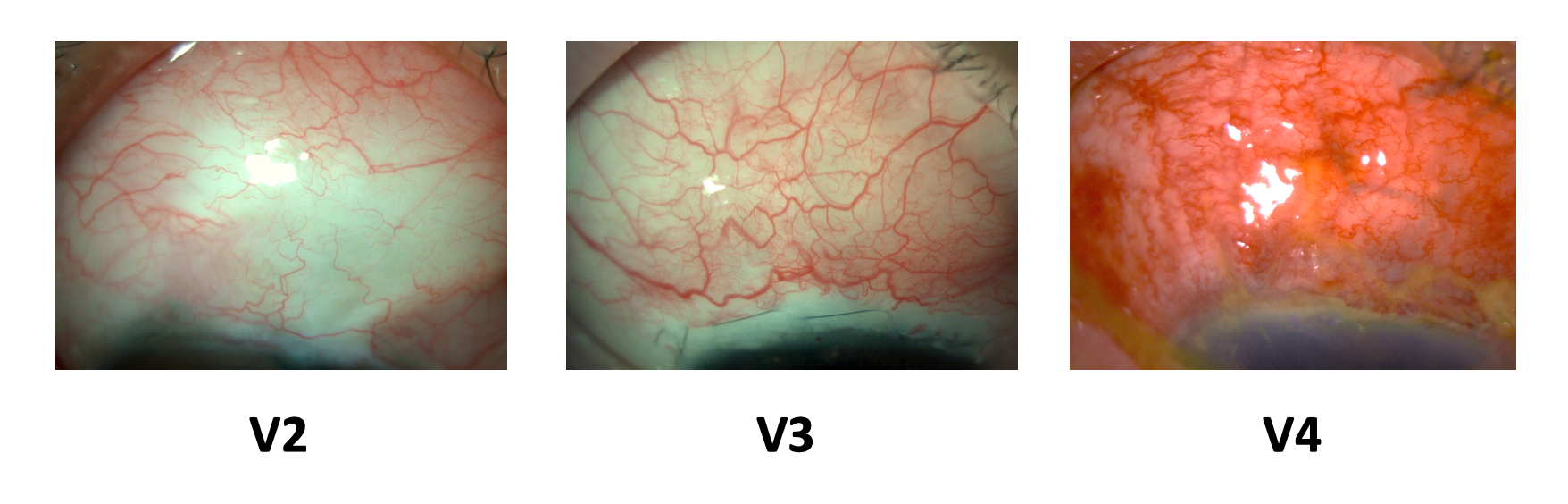

Supplement: S1 Fig — (TIFF) [file pone.0245429.s002.tiff]
